# Attitudes, beliefs and practice of Egyptian healthcare workers towards seasonal influenza vaccination

**DOI:** 10.1111/irv.12868

**Published:** 2021-06-11

**Authors:** Sally Adel Hakim, Wagdy Amin, Mohamed Farouk Allam, Asmaa M. Fathy, Amira Mohsen

**Affiliations:** ^1^ Department of Community, Environmental and Occupational Medicine Faculty of Medicine Ain Shams University Cairo Egypt; ^2^ Ministry of Health and Population, Chest Directorate National Tuberculosis Control Program Cairo Egypt; ^3^ Department of Family Medicine Faculty of Medicine Ain Shams University Cairo Egypt; ^4^ Community Medicine Department National Research Centre Cairo Egypt; ^5^ World Health Organization Country Office Cairo Egypt

**Keywords:** attitude, belief, cross sectional study, Egypt, healthcare workers, influenza vaccination, practice

## Abstract

**Background:**

Seasonal influenza vaccination is highly recommended for healthcare workers (HCWs) every year to protect them and reduce the risk of disease transmission at workplaces. Relatively few studies addressed influenza vaccination in the Eastern Mediterranean Region.

**Objectives:**

The main objective of this study was to explore the attitudes, beliefs and practice of Egyptian HCWs towards seasonal influenza vaccine.

**Methods:**

This is a nationwide cross‐sectional study. Data were collected through self‐administered structured questionnaire. A sample of 3534 HCWs (physicians and nurses) was collected from different levels of healthcare facilities.

**Results:**

The proportion of seasonal influenza vaccine uptake during the last season was 30.7% while the percentage of ever vaccinated was 46.8%. The most identified reason for non‐compliance was lack of trust about vaccine efficacy and its adverse events. Around 80% of participants expressed positive attitude towards influenza vaccine and the vast majority (98%) agreed to uptake the vaccine during pandemic. There was significant positive association between attitude score and influenza vaccine uptake. Raising awareness about vaccine and ensuring vaccine availability were the main suggestions by HCWs to improve vaccine uptake.

**Conclusions:**

Although there was positive attitude towards influenza vaccine, yet vaccination coverage was suboptimal particularly among those working in university hospitals. Educational messages and operational strategies addressing motivators and barriers that emerged from this study are needed to optimize vaccine uptake.

## 1 INTRODUCTION

Globally, annual influenza vaccination rates among healthcare workers (HCWs) are almost low despite recommendations from WHO and national public health institutions of many countries.^1^

Unvaccinated HCWs are an important source of nosocomial influenza. Transmission of influenza virus from patients to HCWs, from HCWs to patients and among HCWs has been well documented.^2^, ^3^ The possible consequences of infected HCWs include increased morbidity and mortality among patients at risk of contracting influenza and a high rate of sickness absenteeism among HCWs themselves resulting in shortage of staff, additional burden on the health system and reduce the healthcare quality.^4^, ^5^

Influenza vaccination is the most effective strategy for the prevention of influenza virus infection and the potentially severe complications. The World Health Organization (WHO) and the Advisory Committee on Immunization Practices (ACIP) recommend that HCWs should be vaccinated annually against influenza. It is also recommended that healthcare organizations implement policies and procedures to encourage HCWs vaccination.^3^ Ahmed and colleagues found that vaccination of HCWs significantly reduced influenza‐like illness and all‐cause mortality among patients.^5^

Relatively, few studies addressed influenza vaccination in the Eastern Mediterranean Region.^6^ In a study among HCWs in three Middle East countries, the vaccination rate was 24.7%, 67.2% and 46.4% in United Arab Emirates, Kuwait and Oman, respectively. Moreover, the different variables associated with the non‐compliance of HCWs to the annual influenza vaccination were lack of time (31.8%) followed by unawareness of vaccine availability (29.4%), unavailability of vaccine (25.4%), doubts about vaccine efficacy (24.9%), lack of information about importance (20.1%) and concerns about its side effects (17.3%).^7^

Recent WHO Strategic Advisory Group of Experts (SAGE) on Immunization for influenza vaccination recommended HCWs as one of the highest priority groups for receipt of influenza vaccines during the current COVID‐19 pandemic.^8^

To our knowledge, this is the first study among HCWs in Egypt tackling influenza vaccination. The purpose of the present nationwide study is to explore attitudes, beliefs and practice of HCWs in Egypt regarding seasonal influenza vaccination. The ultimate goal is to gather data to plan future interventions and policies aiming at increasing influenza vaccination coverage among Egyptian HCWs.

## 2 METHODOLOGY

### 2.1 Study settings

A nationwide cross‐sectional study was conducted between June and October 2019 in 11 governorates representing different country regions. From Middle region, both Cairo and Giza governorates were selected purposively as they include all types of healthcare services and large number of HCWs. From South region, three governorates were randomly selected: Fayoum, Menia and Assiut. From North region, 5 governorates were randomly selected: Qalyoubia, Gharbia, Menoufia, Sharkia and Alexandria while one governorate was selected from Suez Canal zone (Port Saied).

From each selected governorate, the capital city was purposively included to ensure the representation of all healthcare service levels provided by MOHP, in addition to the university hospitals.

### 2.2 Study participants

Physicians and nurses of the selected healthcare facilities were asked to participate in the study. Many specialties were included in the study particularly those working in Intensive Care Units (ICU), Neonatal Intensive Care Units (NICU), chest hospitals and fever hospitals. Also, HCWs in the departments of internal medicine, paediatrics and primary health care were represented in the sample. Eligible subjects were physicians and nurses providing health care to patients, while those with no or minimal contact with patients and those with less than 1‐year in‐job experience were excluded.

### 2.3 Sample size determination

Sample size was calculated using influenza vaccine uptake of 28.2% as estimated in Eastern Mediterranean regions,^6^ using 95% confidence level and 5% margin error. During calculation, we considered the average number of HCWs (physicians and nurses) per governorate = 12 000. Accordingly, the sample size required was 303 HCWs. As the study was conducted in 11 governorates, the total sample size required was =303 × 11 = 3333 HCWs. Using an estimated response rate of 90%, the required sample increased to 3703. Sample size was calculated by the software program Epi Info version 7.0 for Windows. CDC ‐ Atlanta, USA.^9^

### 2.4 Study tool

A structured self‐administered questionnaire was designed to be distributed to target study subjects at their workplaces. The questionnaire included selected socio‐demographics information, in addition to questions on HCWs beliefs, attitudes and practice regarding seasonal influenza vaccination.

Healthcare workers beliefs were assessed using questions on who is mostly in need of influenza vaccine, measures taken in case of exposure to influenza patient, beliefs of vaccine effectiveness and safety, and vaccine acceptability. While the attitude questions were developed using 5 points Likert scale to assess HCWs attitude towards influenza infection severity and complications, having concerns of getting the infection or transmitting influenza to their families, importance of and planning to have the vaccine next season and the need for providing the vaccine free of charge.

Practice questions included ever having influenza vaccine and being vaccinated last season and reasons for getting or rejecting uptake of influenza vaccine.

The tool was piloted and revised based on the answers of 60 HCWs (30 physicians and 30 nurses). Reliability and validity of the tool were assessed by experts in public health and epidemiology before distribution (Cronbach's Alpha was 0.83). Data collectors and field supervisors were trained before starting field visits to healthcare facilities.

### 2.5 Data management and statistical analysis

The data were reviewed for completeness and consistency. Double data entry was performed using Microsoft Excel 2010. The statistical analysis included descriptive statistics as frequency, percentage, odds ratio (OR) and their 95% CI. Attitude questions were analysed using a Likert scoring system of 1‐5, attitude was considered positive if scores were (>75%) of total scores, neutral between (60 and 75%) and negative if scores are (< 60%) of total scores.

For bivariate analysis, statistical comparisons were performed using Pearson's Chi‐square test. Thereafter, univariate and multivariable logistic regression models were applied to identify the predictor variables associated with influenza vaccination uptake during last season. The level of significance was set at *P* < .05. Statistical analyses were performed using the Statistical Package for the Social Sciences (IBM‐SPSS) version 21.0 (IBM Corporation).

## 3 RESULTS

Of 3710 HCWs asked to participate, 3534 responded and filled the questionnaire (response rate = 95.3%) including 1745 were physicians (49.4%). Two thirds of respondents were females, and 71.5% were less than 40 years old. Around 70% of HCWs were affiliated to MOHP healthcare facilities while the rest were working in university hospitals. Table 1 illustrates the demographic characteristics of the study participants.

**TABLE 1:** Socio‐demographic characteristics of study participants (n = 3534)

Characteristics	Category	Frequency	Percentage
Age groups (years)	<30 years	1189	33.6
	30‐39	1341	37.9
	40‐49	653	18.5
	≥50	351	9.9
Gender	Males	1186	33.6
	Females	2348	66.4
Occupation	Doctor	1745	49.4
	Nurse	1789	50.6
Education	Secondary (nurses)	1378	39.0
	University (bachelor)	1182	33.4
	Post University	974	27.6
Type of facility	MOHP (general and district hospitals)	859	24.3
	MOHP‐ Chest hospitals	1078	30.5
	MOHP‐ Fever and infectious diseases hospitals	177	5.0
	MOHP ‐ PHCs	351	9.9
	University hospitals	1069	30.2
Work experience (in years)	1‐5	1171	33.1
	6‐10	783	22.2
	11‐20	967	27.4
	>20	613	17.3
Specialty	Chest	1140	32.2
	Internal medicine	824	23.3
	Intensive care	465	13.1
	Paediatrics	447	12.6
	General practitioner	341	9.6
	Tropical and infectious diseases	190	5.4
	Others (lab, radiology)	127	3.6
Tobacco smoking	Non‐smokers	3247	91.9
	Current smokers	184	5.2
	Ex‐smokers	103	2.9
Chronic illness (any)	Yes	716	20.3
	No	2818	79.3

### 3.1 Vaccination status

Of 3534 respondents, 1653 (46.8%) reported having influenza vaccine at least one time, 342/1653 (20.7%) mentioned they had it once and around 40% gave history of 2 to 4 times uptake of the vaccine (Table 2).

**TABLE 2:** Seasonal influenza vaccination uptake by HCWs and their confidence level towards influenza vaccine

	Category	No.	Percentage
Ever vaccinated before	Yes	1653	46.8
	No	1881	53.2
No. of times of vaccinations before (n = 1653)	Once	342	20.7
	2‐4	663	40.1
	≥5	227	13.8
	Don't remember	421	25.4
Vaccination during last season	Yes	1085	30.7
	No	2449	69.3

**FIGURE 1:**
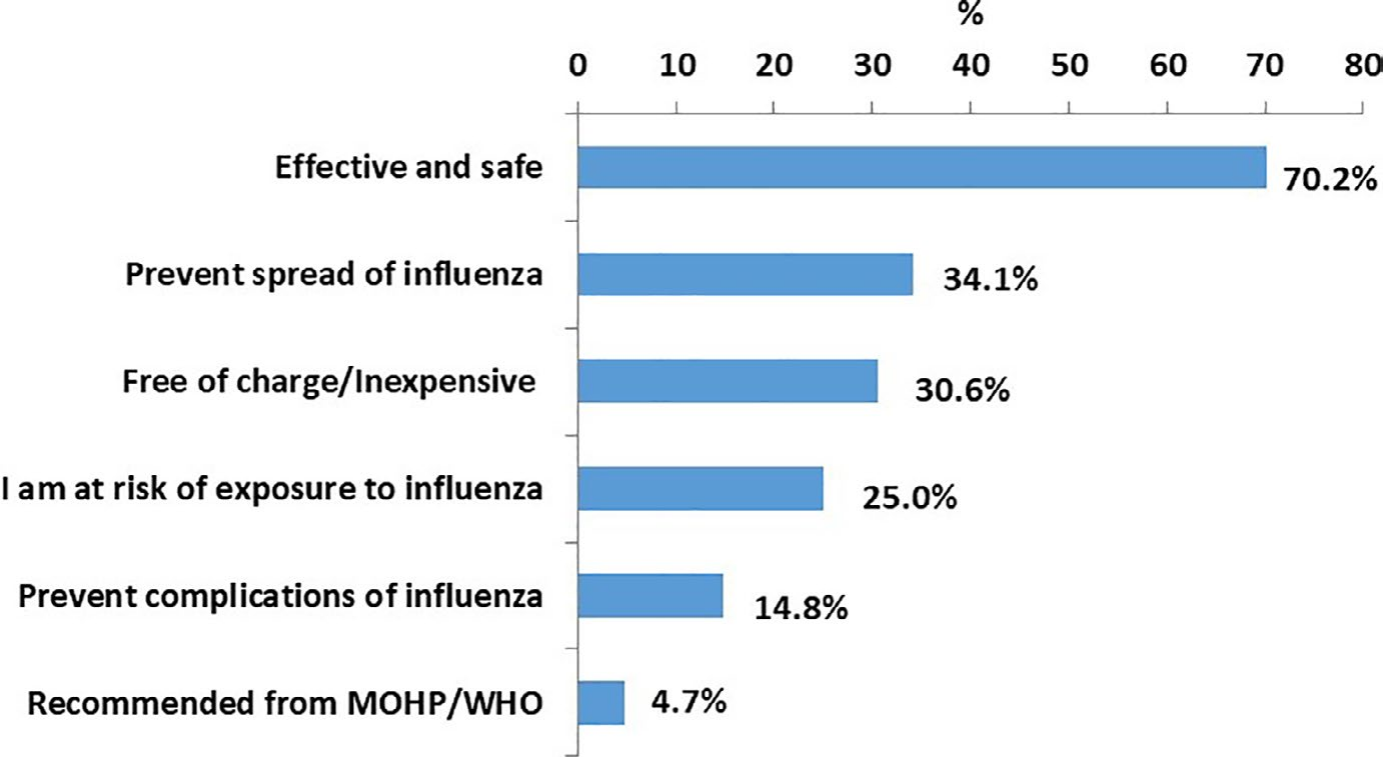
Main reasons of getting the influenza vaccine

Among those who had ever been vaccinated, the main reasons for vaccination were as follows: vaccine is effective and safe (70.2%), it prevents influenza (34.1%), is free of charge (30.6%) and HCWs are at risk of getting influenza infection (25.0%) (Figure 1).

**FIGURE 2:**
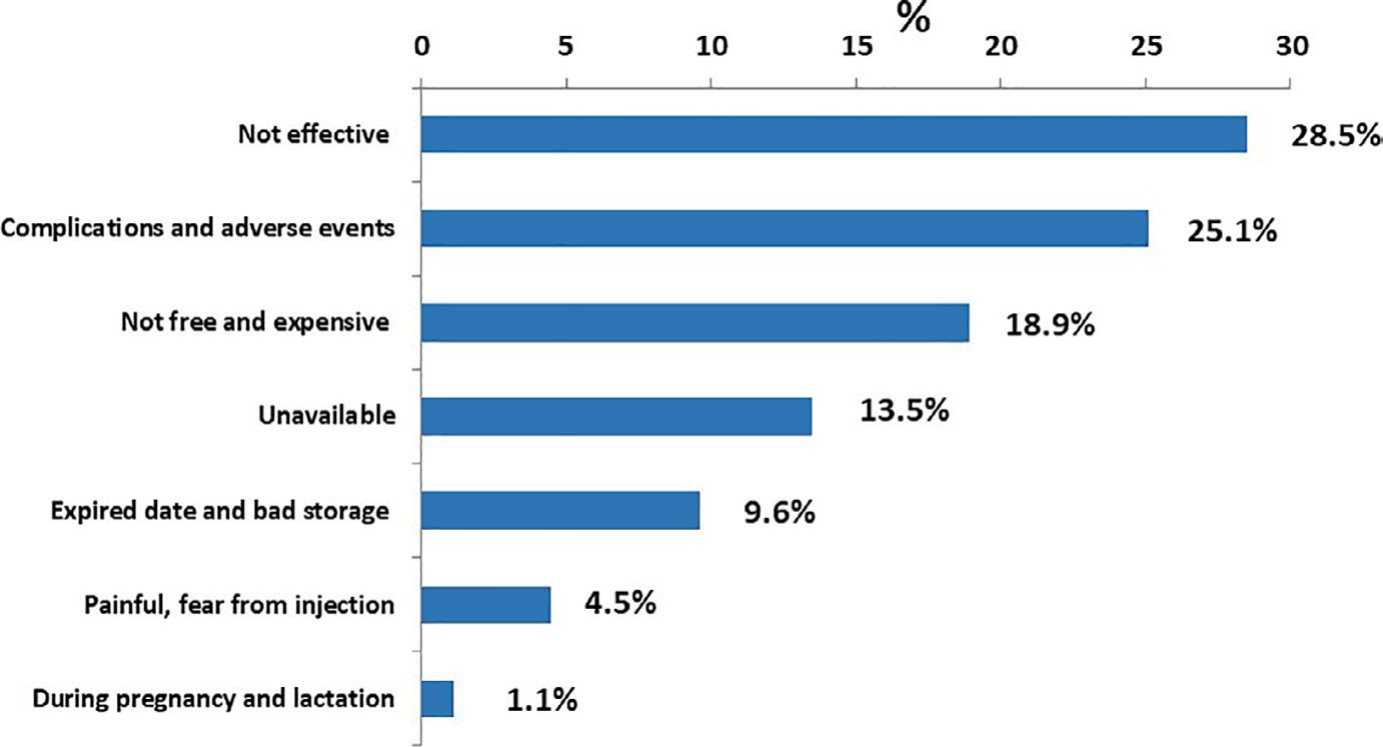
Reasons for rejecting uptake of influenza vaccine among HCWs

While among those who did not get the vaccine during the last season, the main reasons not to get vaccinated were as follows: vaccine is ineffective (28.5%), it causes complications and adverse events (25.1%), and vaccine expenses (18.9%) (Figure 2).

### 3.2 HCWs beliefs and attitude towards influenza vaccination

Although 1292 (36.6%) think that the vaccine is effective in prevention of the disease, yet 2891 (81.8%) agreed to have the vaccine if available and free of charge, and 3463 (98.0%) agree to be vaccinated during pandemics (Table 3). The recommended groups to be vaccinated identified by participants included HCWs (82.2%) elderly people (77.5%), patients with chronic illnesses (68.9%), while only 49.3% identified pregnant women as a target group for influenza vaccination (Table 3). Of all respondents, 84.4% believed that the most effective measure for influenza prevention is frequent hand washing (84.4%) followed by avoid touching eyes, nose and mouth (84.1%) while 73.3% mentioned vaccine as the most effective measure (Table 3).

**TABLE 3:** HCWs beliefs towards Seasonal influenza vaccination

Question	Correct answer	
	No.	%
Vaccination is especially important for		
Healthcare workers	2905	82.2
Elderly	2739	77.7
Pregnant women	1742	49.3
Children aged 6‐59 months	2400	67.9
Individuals with specific chronic medical conditions	2435	68.9
Influenza vaccine is effective and safe	1292	36.6
Most effective measures for influenza prevention at healthcare settings		
Vaccination	2591	73.3
Frequent hand washing	2999	84.9
Avoid touching eyes, nose and mouth	2973	84.1
Avoid direct contact with patients whenever possible	2822	79.9
Wear face mask when in contact with patient	2820	79.8
Vaccination acceptability	No.	%
I agree to take the vaccine if available at workplace and free of charge	2891	81.8
I agree to take the vaccine during pandemics	3463	98.0

**FIGURE 3:**
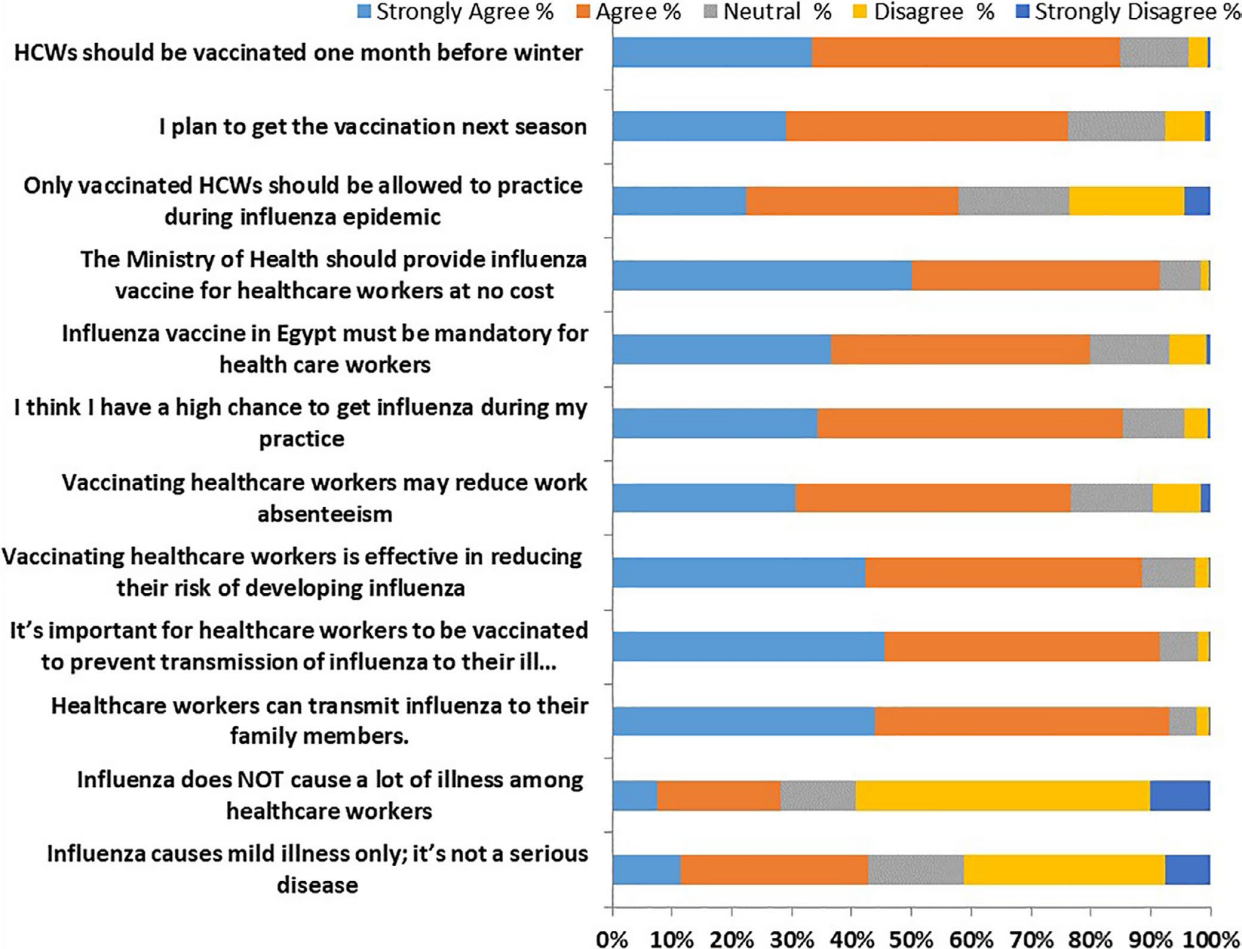
Attitudes of HCWs towards seasonal influenza vaccination

Most of the participants (85.2%) understand that HCWs are at higher risk of getting influenza during work, and most of them realize that it is important to have the vaccine to protect themselves, prevent disease transmission to their patients and families (88.6%, 91.5% and 93.0%, respectively) (Figure 3). Almost half of participants (42.8%) think that influenza is a mild disease and around 28.2% believe that influenza does not cause a lot of serious illness. Most of participants recommend that the vaccination for influenza should be mandatory for HCWs in Egypt and free of charge (79.9% and 91.6%, respectively) and 76.2% are planning to get the vaccine next season. Results showed that 2,823 (79.8%) of HCWs had positive attitude towards influenza vaccination, 635 (18.0%) had neutral attitude and only 76 (2.2%) are having negative attitude (Figure 3).

### 3.3 Predictors of influenza vaccination in HCWs

Overall, 1085 (30.7%) had been vaccinated in the last season (Table 2). Socio‐demographic factors associated with vaccine uptake during the last season revealed that vaccination rate was significantly higher in participants older than 30 years of age, in females, nurses who had secondary school education, working for MOHP health facilities, chest and infectious diseases hospitals, having experience >5 years and specialized HCWs (*P* values <.001) (Table 4).

**TABLE 4:** Association of socio‐demographic characteristics with vaccine uptake in the last season

	Category	Total	Vaccine uptake No. (%)	Unadjusted OR (95% CI)#	*P* value
Age groups (years)	<30 years^®^	1189	189 (15.9)	–	<.001
	30‐39	1341	433 (32.3)	2.52 (2.08‐3.06)	
	40‐49	653	278 (42.6)	3.92 (3.15‐4.89)	
	≥50	351	185 (52.7)	5.90 (4.54‐7.65)	
Gender	Males^®^	1186	326 (27.5)	–	.003
	Females	2348	759 (32.3)	1.26 (1.08‐1.47)	
Occupation	Doctor^®^	1745	465 (26.6)	–	<.001
	Nurse	1789	620 (34.7)	1.46 (1.29‐1.69)	
Education	University grade^®^	1189	226 (19.0)	–	<.001
	Post‐graduate	974	336 (34.5)	2.44 (1.84‐2.73)	
	Secondary nursing school	1371	528 (38.1)	2.69 (2.23‐3.20)	
Type of facility	University hospitals^®^	1069	77 (7.2)	–	<.001
	General and district hospitals$	859	263 (30.6)	5.69 (4.33‐7.47)	
	Chest hospitals$	1078	603 (55.9)	16.36 (12.60‐21.24)	
	Infectious diseases hospitals$	177	98 (55.4)	15.98 (10.97‐23.28)	
	Primary healthcare units$	351	44 (12.5)	1.85 (1.25‐2.73)	
Work experience (years)	1‐5^®^	1171	139 (11.9)	–	<.001
	6‐10	783	237 (30.3)	3.22 (2.55‐4.07)	
	11‐20	967	425 (44.0)	5.82 (4.68‐7.24)	
	>20	613	284 (46.3)	6.41 (5.05‐8.13)	
Specialty	Intensive care	465	133 (28.6)	4.23 (2.21‐8.09)	<.001
	Chest	1140	610 (53.5)	12.14 (6.47‐22.77)	
	Tropical and infectious diseases	190	72 (37.9)	6.44 (3.25‐12.76)	
	Internal medicine	824	137 (16.6)	2.10 (1.10‐4.01)	
	Paediatrics	447	82 (19.3)	2.37 (1.22‐4.60)	
	Family physicians	341	40 (11.7)	1.40 (0.70‐2.82)	
	Others (lab/dermatology/radio‐diagnosis/surgery/)^®^	127	11 (8.7)	–	
Tobacco smoking	Non‐smokers	3247	1000 (30.8)	1.32 (0.84‐2.07)	.445
	Current smokers	184	59 (32.1)	1.40 (0.81‐2.40)	
	Ex‐smokers^®^	103	26 (25.2)	–	

Note

$ = MOHP, ® = Reference group, # = Using univariate logistic regression.

There was a significant positive association between attitudes score and influenza vaccine uptake. HCWs with positive attitudes score were 6.39 times more likely to be vaccinated compared to HCWs with negative score (*P* < .001). Also, HCWs who expressed confidence towards influenza vaccine efficacy were more likely to be vaccinated than non‐confident HCWs (OR = 5.49, 95% CI = 4.29‐7.03). HCWs who suffered from any chronic diseases were more likely to uptake the influenza vaccine (OR = 1.55, 95% CI = 1.30‐1.84) than HCWs with no past history of chronic illnesses (Table 5).

**TABLE 5:** Univariate logistic regression of attitude score, confidence towards influenza vaccine efficacy and history of chronic illnesses with vaccination uptake in the last season

	Category	Total	Vaccine uptake no. (%)	Unadjusted OR (95% CI)#	*P* value
Attitude score	Negative^®^	76	6 (7.9)	–	<.001
	Neutral	635	80 (12.6)	1.68 (0.71‐4.00)	
	Positive	2823	999 (35.4)	6.39 (2.77‐14.76)	
Confident towards vaccine efficacy	Not confident^®^	615	95 (15.0)	–	<.001
	Neutral	1627	358 (22.0)	1.60 (1.25‐2.06)	
	Confident	1292	635 (49.1)	5.49 (4.29‐7.03)	
Chronic Illness (any chronic illness)	No^®^	2818	2008 (71.3)	–	<.001
	Yes	716	441 (61.6)	1.55 (1.30‐1.84)	
Hypertension	No^®^	3127	903 (28.9)	–	<.001
	Yes	407	182 (44.7)	1.99 (1.62‐2.46)	
Diabetes Mellitus	No^®^	3284	977 (29.8)	–	<.001
	Yes	250	108 (43.2)	1.80 (1.38‐2.33)	
Heart Diseases	No^®^	3440	1049 (30.5)	–	.106
	Yes	94	36 (38.3)	1.42 (0.93‐2.16)	
Other chronic diseases (eg renal, COPD etc)	No^®^	3305	1009 (30.5)	–	.339
	Yes	229	76 (33.2)	1.13 (0.85‐1.50)	

Note

#: using univariate logistic regression, ® = Reference group.

The variables that remained significant after performing multivariate logistic regression included nurses who had secondary school education, working at MOHP healthcare facilities, confidence in influenza vaccine effectiveness and safety, positive attitude towards vaccination, work experience >5 years and specialized HCWs (Table 6).

**TABLE 6:** Multivariate logistic regression of socio‐demographic, job characteristics and behavioural factors with vaccination uptake in the last season

Factor	Category	Adjusted OR (95% CI)#	*P* value
Education	University^®^	–	
	Post university	1.07 (0.81‐1.41)	.651
	Secondary nursing school	1.50 (1.10‐2.04)	.011
Type of facility	University hospitals^®^	–	
	General and district hospitals$	5.07 (3.76‐6.85)	<.001
	Chest hospitals$	7.70 (5.74‐10.32)	<.001
	Infectious diseases hospitals$	9.91 (6.30‐15.61)	<.001
	Primary healthcare centre$	2.52 (1.36‐4.65)	.003
Specialty	Others^®^	–	
	Intensive care	5.96 (2.96‐12.00)	<.001
	Chest	6.55 (3.341‐12.84)	<.001
	Tropical & infectious diseases	3.40 (1.57‐7.36)	.002
	Internal medicine	3.00 (1.52‐5.93)	.002
	Paediatrics	2.26 (1.12‐4.55)	.022
	Family physicians	2.26 (0.97‐5.24)	.058
Work experience (years)	1‐5^®^	–	
	6‐10	2.44 (1.85‐3.21)	<.001
	11‐20	3.13 (2.40‐4.10)	<.001
	>20	2.77 (2.06‐3.74)	<.001
Attitude score	Negative^®^	–	
	Neutral	1.68 (0.66‐4.30)	.280
	Positive	3.42 (1.38‐8.50)	.008
Confident towards vaccine efficacy	Not confident^®^	–	
	Neutral	1.57 (1.18‐2.08)	.002
	Confident	3.58 (2.70‐4.75)	<.001

Note

# = using Multivariate logistic regression, ® = Reference group, $ = MOHP facilities.

NB Variables that entered at the beginning of the model but not included in the final model were age groups, gender, profession and chronic diseases.

**FIGURE 4:**
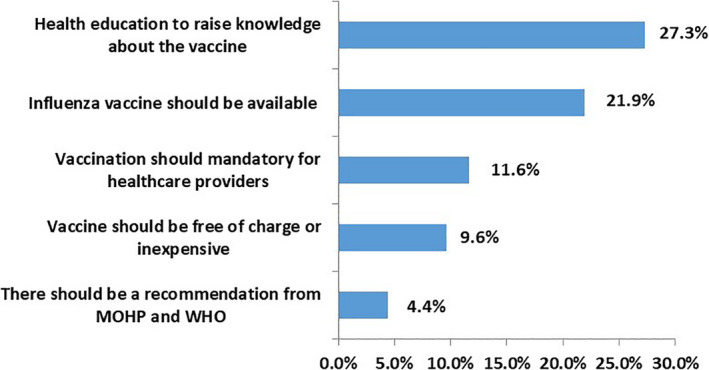
Suggestions from healthcare workers to improve influenza vaccine uptake (N = 3534)

The most common suggestions raised by participants to improve the coverage rate of influenza vaccine uptake among healthcare professionals were health education about influenza vaccine particularly about efficacy and safety was mentioned by 27.3% of the respondents followed by availability of the vaccine at work (21.9%). Other suggestions included mandatory influenza vaccination for all HCWs (11.6%) and offering vaccine free of charge or inexpensive/ reasonably priced (9.6%) (Figure 4).

## 4 DISCUSSION

The present study is the first study to estimate influenza vaccination coverage among Egyptian HCWs and explore their beliefs and attitudes towards seasonal influenza vaccination to improve vaccination coverage among them.

The current study demonstrates a vaccination ever uptake rate among HCWs equal to 46.8% and about 31% in the last season prior to the study. These rates are higher than that reported from EMR (28.2%),^6^ UAE (24.7%),^7^ India (4.4%),^3^ China (6.8%)^10^ and Pakistan (8.84%),^11^ while they are lower than the vaccination uptake among HCWs in Saudi Arabia (67.6%),^12^ and the rates (60% and 80%) reported from developed countries.^13^, ^14^, ^15^, ^16^

The reasons for lower vaccination rate in developing countries could include lack of national vaccination strategies, level of awareness of HCWs towards influenza vaccination and the vaccine cost. This could be supported by the higher vaccination rate encountered in this study among HCWs of chest and infectious diseases hospitals affiliated to MOHP who are provided the vaccine free of charge.

In the present study, higher vaccine coverage was observed among female HCWs than males, and this is similar with literature data.^17^, ^18^, ^19^ Our finding could suggest higher confidence in the efficacy of the vaccine among females compared with males. Moreover, the higher rates of vaccination observed among older age group (≥50 years) in our study was also reported from other studies,^20^, ^21^, ^22^, ^23^, ^24^ a finding that could be explained by longer experience and higher risk of severe infection.

The international literature reports higher vaccination coverage rates of physicians than other HCWs.^14^, ^17^, ^25^ However, the present study showed higher coverage among nurses (34.7%) compared with physicians (27.5%). This is in line with the results of a study from Brazil which showed 69% coverage among nurses versus 49.1% among physicians.^13^ We may relate our results to the higher positive attitude among nurses than physicians. Other studies from Saudi Arabia, Australia and Ireland revealed no significant differences in vaccination coverage among the different professions.^12^, ^15^, ^26^

Higher rates of vaccine coverage were found among staff working in related hospitals (such as chest/fever and infectious diseases hospitals) and specialty (such as chest/tropical and infectious diseases). The main factor underlining this finding is offering the seasonal influenza vaccination free of charge to all HCWs in fever/chest hospitals affiliated to MOHP. Furthermore, HCWs in fever/chest hospitals could have higher perception of risk of exposure to influenza infection than HCWs in other specialties. This is in line with a study by *Black and colleagues* who revealed that coverage was highest among HCWs working in locations where vaccination was required and provided on site at no cost, highlighting the importance of availability of vaccination at workplace.^14^

The current study identified some gaps in the attitudes and beliefs among HCWs in Egypt which included perceiving influenza as a mild disease, a finding reported from other studies.^27^, ^28^ Just as reported in previous studies that 27% to 47% of HCWs think that influenza vaccine could cause post vaccination adverse events,^16^, ^27^, ^29^, ^30^ 25.1% of our respondents shared this misconception. Both findings could explain the suboptimal rate of vaccination observed in our study. In spite that most of participating HCWs agreed that influenza vaccine could be effective in reducing their risk of developing influenza, yet lower percent were planning to get the vaccine next season. Although there is a sound attitude towards influenza vaccination among the participating HCWs, yet it is inconsistent with their practices.

It is noteworthy that about 80% of the participants in our study agreed with the implementation of a compulsory seasonal vaccination strategy in healthcare settings. This issue is under debate in the scientific and public health community.^31^, ^32^ Interestingly, this observation is in disagreement with previous data reporting that mandatory vaccination programmes were in fact badly perceived by European HCWs.^33^

This large representative group of participants allowed us to identify the main reasons that contributed to HCWs' decisions on influenza vaccination. The prevalent role of vaccine safety in determining flu vaccination uptake has been previously reported ^34^, ^35^ and has been identified in the current study as the principle reason for accepting vaccination among those who had ever been vaccinated. Same result was reported in other studies and surveys.^27^, ^36^, ^37^, ^38^ The second powerful motivator in our study was the desire to prevent the spread of infection to others (patients/family/colleagues). Also, other investigators have reported the desire to protect others as the highest rated motive for vaccination.^15^ Nevertheless, many studies have demonstrated that self‐protection and protection of family members and other people close to HCWs are main factors motivating HCWs to receive flu vaccination.^16^, ^18^, ^39^

Low vaccine cost was mentioned by 30% of participants to be a cause of accepting vaccination. Added to that, affording it for free or at low cost was one of their recommendations to improve the vaccine uptake. Also most of our participants were more willing to get vaccinated in the future if the vaccine is offered for free by the government.

Adherence to recommendations by MOHP or WHO was a weak driving factor for vaccine uptake as it was reported by only 4.4% of our participants; however, this was one of the principal reasons for immunization of HCWs in the study by Black and colleagues.^14^

Among the study non‐vaccinated HCWs, one of the major concerns was the perceived lack of efficacy of the vaccine, a finding previously reported in other studies.^27^, ^36^ Furthermore, mistrust of the vaccine storage and expiration date was a reason for rejecting vaccine uptake and is in line with what was reported among European HCWs.^40^ Clarifying misconceptions about vaccine safety and efficacy should play an essential role in any future educational campaigns. The cost of the vaccine has been previously reported as a possible barrier against influenza vaccine acceptance,^41^, ^42^ and was also one of the important reasons for declining vaccine uptake by the participating HCWs. In addition, the unavailability of the vaccine calls for concerted efforts to increase HCWs awareness and increase places where vaccine is available in appropriate quantities and timing.

### 4.1 Strengths and limitations

The strengths of our study are related to the appropriate sample size of HCWs represented from all the regions across the country and covering different levels of healthcare facilities, the high response rate and the different clinical areas represented. We believe that the questionnaire's design was able to capture the real preferences of HCWs.

However, there are some limitations in this study. First, influenza vaccination status was self‐reported by respondents, not subject to independent verification, and potentially influenced by social desirability bias. Recall bias is another potential limitation. Also, this study enrolled HCWs affiliated to governmental sectors (MOHP and university) while those working in private sectors were not included in the study sample, however, most of HCWs in governmental settings also work in private sectors.

## 5 CONCLUSION AND RECOMMENDATIONS

In conclusion, influenza vaccine coverage is suboptimal among HCWs in Egypt. Low rates stem from a variety of reasons ranging from inadequacy of knowledge to misperceptions and fears regarding vaccine efficacy and safety, in addition to high cost and unavailability. These findings should be used to customize and improve any future promotion campaigns, in order to overcome the identified barriers. Also, operational strategies addressing vaccination accessibility at workplace need to be implemented to improve vaccine uptake.

Participants in this study were mostly having positive attitudes and beliefs towards influenza vaccine, and this provides better opportunity to improve vaccine coverage through the tailored health education campaigns and operational strategies which hinder the barriers that limit compliance to vaccination.

## CONFLICT OF INTEREST

None declared.

## AUTHOR CONTRIBUTIONS

**Sally Adel Hakim:** Data curation (equal); Formal analysis (equal); Investigation (equal); Methodology (equal). **Wagdy Amin:** Data curation (equal); Investigation (equal); Methodology (equal). **Mohamed Farouk Allam:** Conceptualization (equal); Data curation (equal); Formal analysis (equal); Investigation (equal); Methodology (equal). **Asmaa Fathy:** Data curation (equal); Formal analysis (equal); Methodology (equal). **Amira Mohsen:** Conceptualization (equal); Data curation (equal); Formal analysis (equal); Funding acquisition (lead); Investigation (equal); Methodology (equal).

## ETHICAL APPROVAL

Verbal informed consent from each participant was obtained before delivering the questionnaire to him/her. We considered filling the questionnaire and returning it back to data collector as a confirmed consent. Each HCW participated in the study was informed about the aim of the research and the voluntary decision to accept or refuse participation. Approval from the Research Ethics Committee (REC) of the Ministry of Health and Population (MOHP)—Central Directorate for Research and Health Development was issued before conducting the study (REC number: FWA00016183) and REC approval of the Faculty of Medicine—Ain Shams University was also obtained. Administrative approvals were obtained before the beginning of the study from the health directorates and the directors of university hospitals.

## Data Availability

The data that support the findings of this study are available from the corresponding author after permission from relevant authority to release data and upon reasonable request.
